# ZRC3308 Monoclonal Antibody Cocktail Shows Protective Efficacy in Syrian Hamsters against SARS-CoV-2 Infection

**DOI:** 10.3390/v13122424

**Published:** 2021-12-03

**Authors:** Pragya D. Yadav, Sanjeev Kumar Mendiratta, Sreelekshmy Mohandas, Arun K. Singh, Priya Abraham, Anita Shete, Sanjay Bandyopadhyay, Sanjay Kumar, Aashini Parikh, Pankaj Kalita, Vibhuti Sharma, Hardik Pandya, Chirag G. Patel, Mihir Patel, Swagat Soni, Suresh Giri, Mukul Jain

**Affiliations:** 1 Indian Council of Medical Research-National Institute of Virology, Pune 411021, India; sreelekshmy88@gmail.com (S.M.); director.niv@icmr.gov.in (P.A.); anitaaich2008@gmail.com (A.S.); 2 Zydus Research Centre, Cadila Healthcare Limited, Ahmedabad 382481, India; sanjeevkumar@zyduscadila.com (S.K.M.); arunsingh@zyduscadila.com (A.K.S.); sanjaybandyopadhyay@zyduscadila.com (S.B.); aashiniparikh@zyduscadila.com (A.P.); pdkalita@zyduscadila.com (P.K.); vibhuti.sharma@zyduscadila.com (V.S.); hardik.pandya@zyduscadila.com (H.P.); chiraggpatel@zyduscadila.com (C.G.P.); Mihir.patel@zyduscadila.com (M.P.); Swagat.soni@zyduscadila.com (S.S.); sureshgiri@zyduscadila.com (S.G.); mukul.jain@zyduscadila.com (M.J.); 3 Department of Neurosurgery, Command Hospital (Southern Command), Armed Forces Medical College (AFMC), Pune 411040, India; paraeagles@gmail.com

**Keywords:** ZRC3308, SARS-CoV-2, monoclonal antibody, prophylaxis, hamsters

## Abstract

We have developed a monoclonal antibody (mAb) cocktail (ZRC-3308) comprising of ZRC3308-A7 and ZRC3308-B10 in the ratio 1:1 for COVID-19 treatment. The mAbs were designed to have reduced immune effector functions and increased circulation half-life. mAbs showed good binding affinities to non-competing epitopes on RBD of SARS-CoV-2 spike protein and were found neutralizing SARS-CoV-2 variants B.1, B.1.1.7, B.1.351, B.1.617.2, and B.1.617.2 AY.1 in vitro. The mAb cocktail demonstrated effective prophylactic and therapeutic activity against SARS-CoV-2 infection in Syrian hamsters. The antibody cocktail appears to be a promising candidate for prophylactic use and for therapy in early COVID-19 cases that have not progressed to severe disease.

## 1. Introduction

Since the first report of occurrence of SARS-COV-2 infection in China on 30 December 2019, the virus has spread rapidly worldwide, accounting for more than 200 million cases and 4 million deaths as of 9 August 2021 [1]. Even though more than twenty vaccines have been granted Emergency Use Authorization (EUA) in multiple countries, the vaccination rate is skewed globally due to the limited accessibility to low- and middle-income countries [2,3]. Due to the inequitable access of vaccines and emergence of new variants with immune escape property, the development of herd immunity may take years [3]. Therefore, there remains an unfulfilled need for therapeutic agents to restrict the morbidity and mortality. Convalescent plasma transfusion has been used as a therapeutic option in the case of novel viral diseases such as Ebola virus disease, SARS, MERS, and coronavirus disease 2019 (COVID-19), but the variability in the donor antibody titers and risk of blood borne diseases remained hurdles [4,5,6,7]. Monoclonal antibody (mAb)-based therapy became an alternative to overcome the limitations of convalescent plasma therapy [8]. The favorable safety profiles and comparatively lesser time for generation and approval made mAbs a feasible therapeutic alternative in case of many emerging disease threats [9].

Monoclonal antibodies can serve as an adjunct to the prophylactic strategy for COVID-19 infections in high-risk groups such as aged and immunocompromised people who have suboptimal responses to vaccination [10]. The techniques of combinatorial display libraries, humanized mice, and B-cell isolation methods have aided in the rapid recovery of many antiviral mAbs [11]. Palivizumab was the first mAb to be approved for treatment against Respiratory Syncitial Virus in 1998 [12]. In 2020, a combination of three mAbs, atoltivimab, maftivimab, and odesivimab-ebgn, was approved by the United States Food and Drug Administration (USFDA) for Zaire Ebola virus therapy [13,14]. More than 200 research laboratories across the world are working on developing highly potent recombinant human mAbs against SARS-CoV-2 to provide an unlimited supply of high-quality product to patients [9]. USFDA has granted EUA for a few of the anti-SARS-CoV-2 mAbs-targeting epitopes on the receptor-binding domain (RBD) of the S protein such as bamlanivimab plus etesevimab, casirivimab plus imdevimab, and sotrovimab for treatment of mild to moderate COVID-19 [15,16,17].

We have developed a cocktail (ZRC-3308) of two highly potent, neutralizing, humanized mAbs, ZRC3308-A7 (CAS RN: 2640223-84-1) and ZRC3308-B10 (CAS RN: 2640224-48-0), that bind with nanomolar affinities to non-competing epitopes on the RBD of the spike protein of SARS-CoV-2. Both of these mAbs were designed to have reduced immune effector functions and increased circulation half-life. Here, we describe the in vitro biological properties of the mAbs and in vivo evaluation against SARS-CoV-2 infection in a Syrian hamster model.

## 2. Materials and Methods

### 2.1. Ethics Statement

The study was approved by the Institute Animal Ethics committee, ICMR-National Institute of Virology, Pune (Approval no: NIV/IAEC/2021/MCL/04, 8 January 2021). The study was performed according to the guidelines of the Committee for the Purpose of Control and Supervision of Experiments on Animals, Government of India.

### 2.2. mAb (ZRC3308-A7 and ZRC3308-B10) Generation

The codon-optimized, light, and heavy chain genes of ZRC3308-A7 (CAS RN: 2640223-84-1) and ZRC3308-B10 (CAS RN: 2640224-48-0) carrying mutations in heavy chain gene at position, leu234Ala, leu235Ala, and Pro329Gly for reduced effector functions and Met428Leu and Asn434Ser for increased half-life, were cloned in a dual assembly eukaryotic expression vector, DGV-GS, with a glutamine synthase selection marker (DGV-GS, Lonza, Basel, Switzerland). Separate DGV-GS vectors (linearized by PvuI enzyme) encoding the light and heavy chain genes of ZRC3308-A7 and ZRC3308-B10 were used for transfection in the CHOK1SVGSKO (Lonza, Basel, Switzerland) cell line by electroporation following pre-optimized parameters using the neon transfection system (Invitrogen). Post transfection, the transfectant pools were subjected to selection in the presence of different concentrations (0, 12.5, 25 µM) of Methionine sulphoximine (MSX) (Sigma Aldrich, St. Louis, MO, USA) in Pro-CHO-5 media (Lonza, Basel, Switzerland).

Cells from the high-expressing transfection pools of ZRC3308-A7 (25 µM MSX) and ZRC3308-B10 (12.5 µM MSX) were plated over Clona Cell CHO CD medium (StemCellTM Technologies, Vancouver, BC, Canada) supplemented with Clone detect Human IgG H + L specific, fluorescein (Molecular Devices, San Jose, CA, USA), for clonal selection using ClonePix2 (Molecular Devices, San Jose, CA, USA). After 7–10 days, based on high exterior mean fluorescent intensity, selected clones were aspirated and plated in 96-well plates and expanded further. Pools/clones were subjected to fed-batch cultures to generate material for in vitro studies. The cells were seeded (0.3 million/mL) in Acti-Pro production medium (Hyclone, GE, Marlborough, MA, USA) in culti-tubes (TPP, Trasadingen, Switzerland) and incubated in a humidified shaker (Kuhner, Birsfelden, Switzerland; 37 °C, 5% CO_2_, and 230 RPM). The cells were cultured for a period of 12–15 days with a daily feed of pre-optimized concentration of Cell Boost 7a and 7b supplements (Hyclone, GE, Marlborough, MA, USA) starting from Day 3. The antibody concentration in cell culture supernatants was estimated by Protein A (MabSelect sure; GE life sciences, now Cytivia, Marlborough, MA, USA) high-pressure liquid chromatography. Cell culture harvests were purified by the Protein A gravity column.

For large-scale production of the two mAbs, the above-mentioned process was scaled up to 20 L and then 200 L in suspension bioreactors using a Fed-batch process and utilizing the previously described production media and feeds. The mAbs were purified from the harvested supernatants using a set of purification steps including MabSelect sure (rProtein A) resin (GE life sciences, now Cytivia), a hydrophobic interaction chromatography (GE lifesciences, now Cytivia), and nano-filtration (Merck MilliPore, Danvers, MA, USA). These purified bulk mAbs were then mixed 1:1 (as per protein content) to generate the cocktail ZRC3308, which was used for all the in vitro and in vivo studies.

### 2.3. Surface Plasmon Resonance (SPR) Analysis

Binding kinetics and affinities towards RBD protein, S Trimer protein, rhFcRn, and recombinant human FcγRIIIa-Phe of the individual mAbs ZRC3308-A7, ZRC3308-B10, and cocktail was assessed using Surface Plasmon Resonance technology on a ProteOn XPR 36 instrument (Bio-Rad, Hercules, CA, USA) using a GLC sensor chip. Filtered and degassed PBST (10 mM phosphate buffer, 150 mM NaCl, 0.005% Tween 20, pH 7.4) was used as a running buffer for all the assays except for the rhFcRn-binding assay, where the PBST buffer with pH 6.0 was used. All the reactions were carried out at 25 °C. RBD protein (Wuhan strain; Cat No: SPD-C82E9; Make: Acro Biosystems, Newark, DE, USA) and S Trimer protein (prefusion state stabilized spike protein of Wuhan strain; Cat No: SPN-C52H8; Make: Acro Biosystems, Newark, DE, USA) were immobilized on the sensor chip surface in two different channels using the standard amine coupling chemistry. To measure the affinity constant (KD) derived from the association rate constant (ka) and dissociation rate constant (kd) for the immobilized proteins, five dilutions of mAbs (individual and cocktail) were prepared in PBST running buffer and injected at a flow rate of 50 µL/min with an association time of 300 s and dissociation time of 600 s. At the end of each cycle, the chip surface was regenerated using 18 s injection of 4 M MgCl_2_. Using the ProteOn Manager Software v 3.1.0.6, the parameters were obtained by fitting the double-reference subtracted data to a 1:1 binding model.

The rhFcRn receptor (Cat: CT009-H08H; Sino Biologics, Beijing, China) was immobilized on a GLC chip using amine coupling chemistry. To measure the ka and kd five dilutions of each of the two ZRC3308mAbs were prepared and injected at a flow rate of 100 µL/min with an association time of 180 s and dissociation time of 600 s. After each sample run, the chip surface was regenerated using PBST, pH 7.4. The rhFcγRIIIa-Phe receptor (Cat: 10389-H27H, Sino Biologics, China) was immobilized on a GLC sensor chip surface using a standard amine coupling chemistry. To measure the ka and kd, five dilutions of each of the two ZRC3308 mAbs were prepared and injected at a flow rate of 100 µL/min with an association time of 240 s and dissociation time of 600 s. The data in the form of sensograms were analyzed using the data-fitting programs available with the ProteOn system.

### 2.4. Pair-Wise Epitope Binning

Epitope binning analysis for the two mAbs was carried out using the ProteOn XPR36 instrument to confirm that the two antibodies do not compete with each other for binding to RBD and therefore bind to unique epitopes on RBD. The RBD protein (Cat No: SPD-C82E9; Make: Acro Biosystems, Newark, DE, USA) was immobilized on a GLC sensor chip surface using standard amine coupling chemistry. A total of 10 mM phosphate buffered saline (PBS) was used as the running buffer. Initially, ZRC3308-A7 and ZRC3308-B10 were run at saturating concentrations (at 50 nM) in different channels immobilized with RBD. In the next run, ZRC3308-A7 and ZRC3308-B10 were individually run at 50 nM over both ZRC3308-A7and ZRC3308-B10 captured channels, and binding patterns were analyzed.

### 2.5. RBD–ACE2 Binding Inhibition

The inhibition potential of the mAbs was assessed using a competitive ELISA. Briefly, ACE-2 protein (Acro Biosystems, Newark, DE, USA) was coated onto ELISA plates (Greiner, Frickenhausen, Germany) at a 1 µg/mL concentration in PBS and was incubated (for 12–72 h) at 2–8 °C in a humid chamber. The plate was then blocked using a 2.0% solution of BSA (SD Fine Chem, Mumbai, India). Different concentrations of ZRC-3308-A7 (1500 to 2.059 ng/mL, three-fold serial dilutions) and ZRC3308-B10 (800 to 3.277 ng/mL, 2.5-fold serial dilutions) were incubated with 100 ng/mL of Biotinylated RBD (Acro Biosystems, Newark, DE, USA) on a plate shaker at room temperature for 60 min to allow the binding of the mAbs to RBD. This mixture was then loaded onto an ACE-2 coated plate to allow the unbound RBD to bind to the coated ACE2. Detection was accomplished using peroxidase conjugated streptavidin at 1/50 K dilution. TMB was used as a substrate (Sigma Aldrich, St. Louis, MO, USA). The reaction was terminated using 1 N sulfuric acid, and the absorbance was read at 450 nm in a multi-mode plate reader (Molecular devices, San Jose, CA, USA).

### 2.6. C1q-Binding Assay

A chemiluminescence-based ELISA method was used to analyze the C1q-binding properties of both ZRC3308A7 and ZRC3308-B10. Plates were coated using different optimal concentrations (350 to 2.116 µg/mL) of ZRC3308-A7 or ZRC3308-B10 or Bryxta (a biosimilar version of bevacizumab, Zydus Cadila) as wildtype IgG1 (60 µg/mL to 0.363 µg/mL) and was incubated in 37 + 2 °C in incubator for 2 h. The coated plates were then blocked using 2% skimmed milk (Hi Media, Mumbai, India). This was followed by the addition of 8 µg/mL C1q (Sigma Aldrich, St. Louis, MO, USA) to the plate wells. Detection was accomplished using 1/1000 diluted peroxidase conjugated sheep anti-C1q polyclonal antibody (Abcam, Cambridge, UK). Subsequently Femto substrate (Thermo Scientific, Waltham, MA, USA) was added, and post 5 min incubation in the dark, luminescent signals were quantified using a multimode reader (Molecular devices, San Jose, CA, USA) in luminescence mode. The four-parameter fit was used to determine the EC_50_ concentrations.

### 2.7. Pseudovirus-Based Neutralization Assay

HEK 293 ACE2-expressing cells (Scripps institute) were used for the assay. Pseudovirus (an HIV-based luciferase expressing lentivirus pseudotyped with SARS-CoV-2 full-length S protein) was obtained from Creative Biogene. A one-step luciferase assay kit from BPS Bioscience was used for detection. The IC_50_ was defined as the dilution of serum at which the relative light units (RLUs) were reduced by 50% compared with the virus control after subtraction of the background RLUs of the cell control.

In brief, the antibody preparations were diluted to a starting concentration of 1 mg/mL and serially diluted with 10-fold dilutions to generate a concentration range of 1 × 10^6^ to 0.001 ng/mL. Sera from SARS-CoV-2-infected individual and Bryxta (Zydus Cadila, Ahmedabad, India) at a concentration of 1 × 10^4^ ng/mL were included as positive and negative controls, respectively.

The pseudovirions were diluted in assay medium to achieve an MOI of 50 per well. Pseudovirions were added to the serially diluted antibody preparations, and the plate was incubated for 60 min at 37 °C in a 5% CO_2_ incubator. Simultaneously, HEK 293 ACE2-expressing cells were trypsinized and counted. HEK 293 ACE2-expressing cells (1.0 × 10^4^ per well) were seeded in a flat tissue culture plate. After completion of incubation, the mixture containing antibody and pseudovirions was added to the seeded HEK 293 ACE2-expressing cells, and the plate incubated for 24 h at 37 °C in a 5% CO_2_ incubator. Following 24 h of incubation, the virus-containing media was replaced with fresh growth medium, and the plate was further incubated for 24–48 h at 37 °C in a 5% CO_2_ incubator. The plate was observed for confluency and, after reaching 85–90% confluency, it was removed to measure the luminescence. A total of 100 µL of firefly luciferase substrate was added to all the wells, and the plate was incubated on shaking condition at room temperature for 30 min to check the transduction efficacy. The plate was read using a luminescence microplate reader (SpectraMax^®^ i3x, Molecular devices, San Jose, CA, USA). The obtained data were normalized against the cell control well. The average of four replicates of percent neutralization data in relation to the virus-only infection control was plotted in a graph against the concentrations of mAbs to obtain the IC_50_ values for the samples. The IC_50_ values were calculated using GraphPad Prism V-9.2.0 (GraphPad Prism, Inc., San Diego, CA, USA).

### 2.8. SARS-CoV-2 Live Virus Plaque Reduction Neutralization Test

SARS-CoV-2 isolate belonging to B.1 lineage (GISAID accession no. EPL_ISL_420546) was used. Vero E6 (ATCC) cells (1.0 × 10^6^ per well) were seeded to 24-well plates in maintenance medium for 24 h at 37 °C in a 5% CO_2_ incubator. The next day, mAbs were serially diluted in assay medium (range of 1 × 10^6^ ng/mL to 0.001 ng/mL). SARS-CoV-2 virus was added to each dilution at 0.01 MOI except the cell controls. The mixtures of virus and antibody were incubated for 60 min at 37 °C in a 5% CO_2_ incubator. Following completion of incubation, the virus and antibody mixtures were added to the pre-seeded Vero E6 cells by first discarding the supernatant followed by replacement with a medium containing 2% carboxy methylcellulose. The plates were incubated for a further 72 h in a CO_2_ humidified incubator at 37 °C. Post-incubation, the cells were fixed with 4% formaldehyde, and the plaques were enumerated by staining with crystal violet stain. The number of plaques was counted, and the percentage inhibition was calculated in comparison to the number of plaques obtained in the wells that contained only the virus but no antibody (positive control). IC_50_ was calculated using GraphPad Prism V-9.2.0 (GraphPad Software, Inc., San Diego, CA, USA). Samples were run in quadruplicate, and averages were plotted on the graph. Further, we performed PRNT_50_ using other SARS-CoV-2 variants such as B.1.1.7, B.1.351, B.1.617.2, and B.1.617.2 AY.1, as described earlier [18].

### 2.9. Pharmacokinetic Study in Hamsters

A total of 18 female hamsters, aged 11–12 weeks, were divided into four groups according to the dose of the cocktail, l i.e., 50 mg/kg (25 mg/kg of ZRC3308-A7 + 25 mg/kg of ZRC-3308-B10), 5 mg/kg (2.5 mg/kg of ZRC3308-A7 + 2.5 mg/kg of ZRC3308-B10), 1.0 mg/kg (0.5 mg/kg of ZRC3308-A7 + 0.5 mg/kg of ZRC3308-B10), and placebo consisting of five animals each, except for the placebo, which consisted of only three animals. Cocktails of ZRC3308-A7 and ZRC3308-B10 were administered via the intraperitoneal (I.P.) route, and the total duration of the study was seven days. Blood was withdrawn from the animals before administration of mAb and at 24, 72, 120, and 168 h post-administration. The serum was separated to assess the concentration of each of the two mAbs by ELISA. For the placebo group, samples were collected only before administration and at 168 h.

### 2.10. ELISA-Based Detection of mAb Levels in Serum

Two separate ELISA methods were used to detect ZRC3308-A7 and ZRC3308-B10 antibodies. In both the immunoassays, the coating reagent used was SARS-CoV-2 S1 protein (Acro Biosystems, Newark, DE, USA). Post 24–72 h of coating and incubation in a humidified chamber at 2–8 °C, ELISA plates (Greiner, Frickenhausen, Germany) were blocked using 2% BSA (SD Fine Chem, Mumbai, India). Subsequently, the calibration curve (ranging from 25 to 0.195 ng/mL) was prepared separately for ZRC3308-A7 and ZRC3308-B10 with pooled, naive, hamster serum diluted 500 times in 0.1% BSA in PBST, and the serum samples containing ZRC-3308 cocktail were added at appropriate dilutions. Detection was accomplished using 1/100,000 diluted peroxidase conjugated goat anti-human lambda light chain secondary antibody for ZRC3308-A7 (Novus Biologics, Centennial, CO, USA) and 1/135,000 diluted Goat anti-Human kappa light chain secondary antibody for ZRC3308-B10 (Novus Biologics, Centennial, CO, USA) to specifically detect the two antibodies. TMB was used as a substrate. The reaction was stopped using 1N sulfuric acid, and the ELISA plates were read in a multi-mode reader (Molecular devices, San Jose, CA, USA) at 450 nm.

### 2.11. Challenge Study in Syrian Hamsters

A total of 60 female Syrian hamsters, aged 7–10 weeks, were used for the prophylactic study, which were randomly divided into five groups of 12 animals each. The groups were high (50 mg/kg), medium (5 mg/kg), and low (1 mg/kg) doses of mAb cocktail, Vivitra (a biosimilar trastuzumab, Zydus Cadila), as an IgG1 isotype antibody (50 mg/kg) control and a placebo control. The animals were intranasally inoculated with 0.1 mL of 10^5.5^ TCID50 of SARS-CoV-2 strain isolate belonging to the B.1 lineage (GISAID accession no. EPL_ISL_420546). The cocktail of mAbs was administered by intraperitoneal injection 2 days before virus infection for the prophylactic group. To the placebo group, only the SARS-CoV-2 intranasal virus challenge was performed without any antibody treatment.

There were three sub-studies in the therapeutic arm. For the first therapeutic study, 60 female hamsters aged 7–10 weeks were used, which were randomly divided into five groups as mentioned for prophylactic study. The mAb treatment was given intraperitoneally at 24 h post-intranasal virus infection. The animals were closely observed for any mortality or clinical signs post administration such as activity, ruffled fur, discharges from natural orifices, labored breathing, and body weight changes. Four animals each of the prophylactic and therapeutic study group were sacrificed by overdose of isoflurane anesthesia on 3, 5, and 7 DPI, and necropsy was performed to collect nasal wash, nasal turbinates, lungs, and blood samples. The serum was separated and stored at −20 °C to test for the level of mAb levels. The organ samples were stored at −80 °C for viral load estimation and in 10% neutral buffered formalin for histopathological analysis. The proportion of the lungs-to-body-weight ratio was also determined.

In the second study of the therapeutic arm, 18 female hamsters aged 8–10 weeks were used, and mAb treatment was given intraperitoneally 6 h post-infection with an intranasal virus (GISAID accession no. EPL_ISL_420546) challenge dose of 10^5.5^ TCID50. The 50 and 5 mg/kg mAb dose groups along with a placebo group with six animals each were included in the study. On Days 3 and 5, three hamsters from each group were sacrificed to assess and collect organ and nasal wash samples.

In the third therapeutic study, a challenge virus (GISAID accession no. EPL_ISL_420546) dose of 10^3.5^ TCID50 was given intranasally, followed by intraperitoneal mAb treatment at 24 h post infection. Fifteen female hamsters aged 16–18 weeks were used for the study. The 50 and 5 mg/kg mAb dose groups along with a placebo group with five animals each were included in the study. The hamsters were sacrificed on Day 5 to assess lung pathology and viral load in organs.

### 2.12. Viral RNA Quantification

Nasal wash samples collected in 1 mL viral transport medium (Himedia, Mumbai, India) and weighed organ samples homogenized in 1 mL media (Sigma Aldrich, St. Louis, MO, USA) were used for RNA extraction. MagMAX™ Viral/Pathogen Nucleic Acid Isolation Kit (Thermo Fisher Scientific, Waltham, MA, USA) was used as per the manufacturer’s instructions. E gene real-time RT-PCR was performed using published primers [19]. SgRNA levels were also estimated by primers targeting the N gene of SARS-CoV-2 using published primers [20].

### 2.13. Histopathological Examination

Lung samples collected were fixed in 10% neutral buffered formalin and were further processed for paraffin embedding and block making following dehydration and clearing. Four to five micron thick sections were made using an automated microtome (Leica, Wetzlar, Germany) and were stained by hematoxylin and eosin staining. The lesions were blindly scored based on the vascular changes, bronchial lesions, alveolar pathological changes such as septal thickening, pneumocyte hyperplasia, consolidation, edematous changes, and inflammatory cell infiltration. Each lesion was scored on a scale from 0 to 4, and the cumulative scores were represented in a graph.

### 2.14. Statistical Analysis

Graphpad Prism version 8.4.3 software was used for data analysis of the experimental challenge study. A Kruskal–Wallis test followed by a Mann–Whitney test was used. *p* < 0.05 was considered to be statistically significant.

## 3. Results

### 3.1. Binding of ZRC3308 mAbs to RBD and Spike Protein Trimer

**Figure 1:**
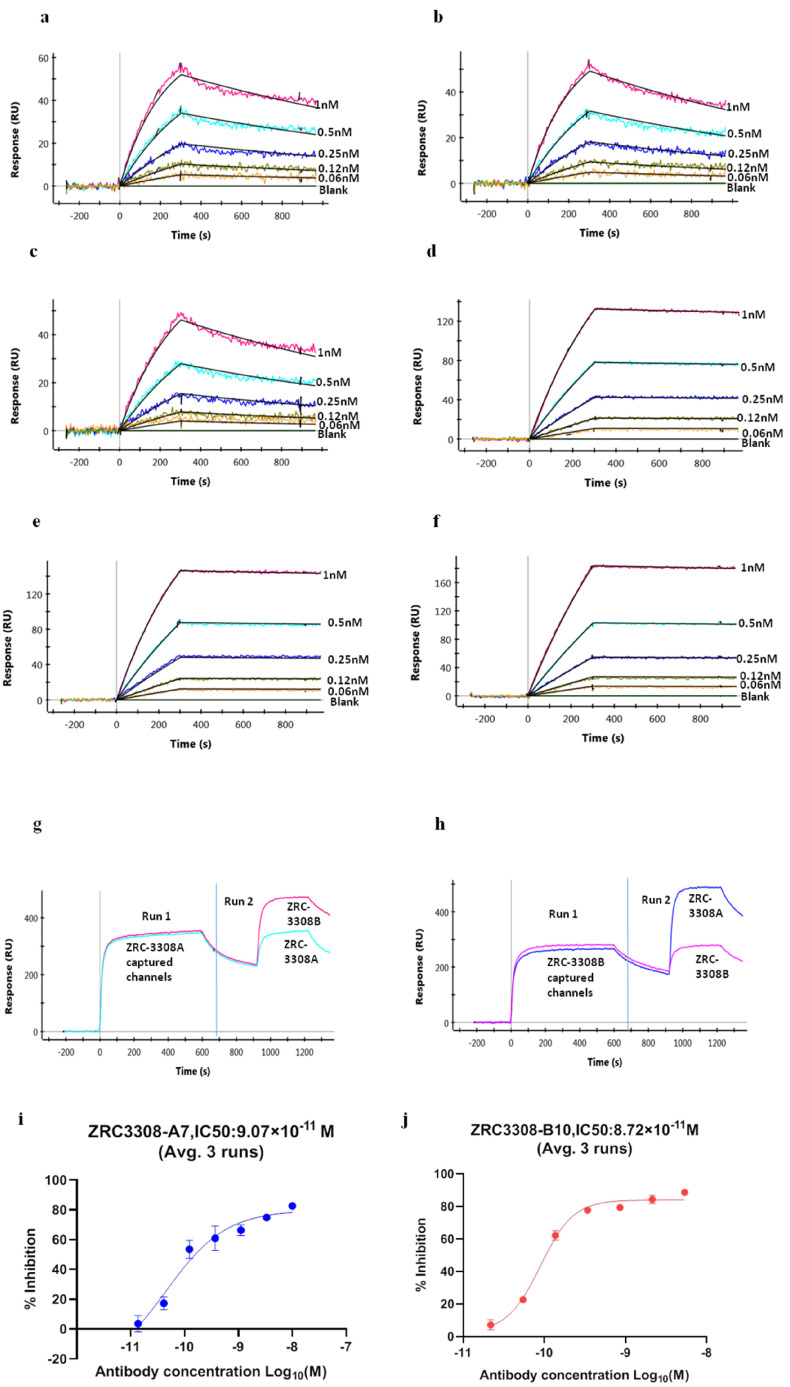
Binding of ZRC3308 to RBD and Spike protein trimer. Binding sensograms of the (**a**) ZRC3308-A7, (**b**) ZRC3308B10, and (**c**) ZRC3308 cocktail to RBD protein and the (**d**) ZRC3308-A7, (**e**) ZRC3308-B10, and (**f**) ZRC3308 cocktail to S protein. (**g**) Binding of ZRC3308-B10 on ZRC3308-A7 precaptured channels and (**h**) binding of ZRC3308-A7 on ZRC3308-B10 precaptured channels. Inhibition of RBD binding to ACE2 in the presence of (**i**) ZRC3308-A7 and (**j**) ZRC3308-B10 represented as mean ± standard deviation of three runs.

The ZRC-3308 cocktail showed similar binding profiles to RBD and S Trimer protein when compared with its individual components, ZRC3308-A7 and ZRC3308-B10 (Figure 1a–f). The binding to both RBD and S trimer protein were with high affinities in the sub nM ranges for both the individual mAbs and their cocktail (Table 1). A pair-wise epitope binning experiment was performed to determine whether each of the two mAbs of the cocktail would bind to RBD even in the presence of the other one. The binding of each antibody to RBD was assessed after the other mAb had been allowed to bind first. Binding of ZRC3308-B10 was observed on ZRC3308-A7 precaptured channels and vice versa, indicating that both the antibodies bind to distinct epitopes on RBD of spike protein as depicted in sensograms in terms of response units (RU) vs time (Figure 1g,h).

The binding of the mAbs to the RBD of SARS-CoV-2 S1 protein is expected to inhibit the interaction of S1 protein to the ACE-2 receptor. The inhibition of RBD binding to ACE-2 in the presence of ZRC-3308 was measured in terms of 50% inhibitory concentration (IC_50_) by plotting four-parameter fit curves of antibody concentration vs. % inhibition, as shown in Figure 1i,j. Both the mAbs were able to bind to the spike protein RBD and inhibit its binding to the ACE2 receptor at sub nM IC_50_ concentrations. The mean IC50 values (Average ± SD) from three runs were observed to be 9.07 ± 1.86 × 10^−11^ [M] and 8.72 ± 0.74 × 10^−11^ [M], respectively, for ZRC3308-A7 and ZRC3308-B10. Convalescent sera showed an inhibition of ~92% all the runs. No inhibition of RBD binding to ACE2 was observed with the human IgG negative control.

### 3.2. Binding of ZRC3308 to Recombinant Human Neonatal Fc Receptor (rhFcRn), FcγRIIIa-Phe, and C1q

**Figure 2:**
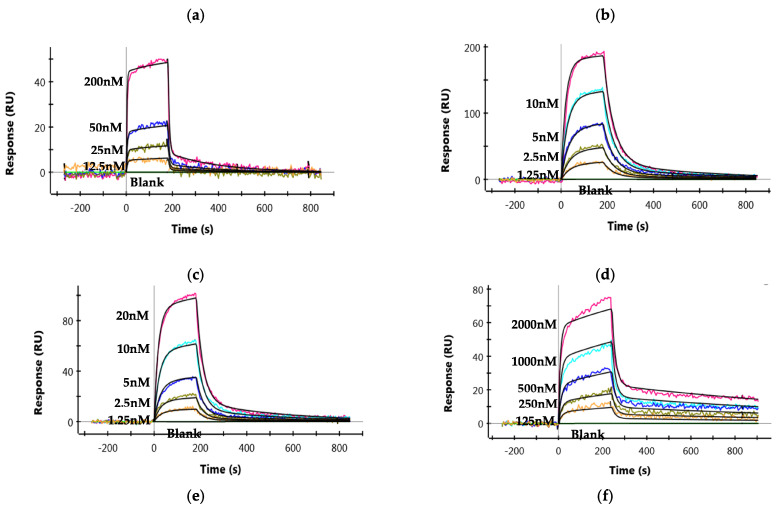

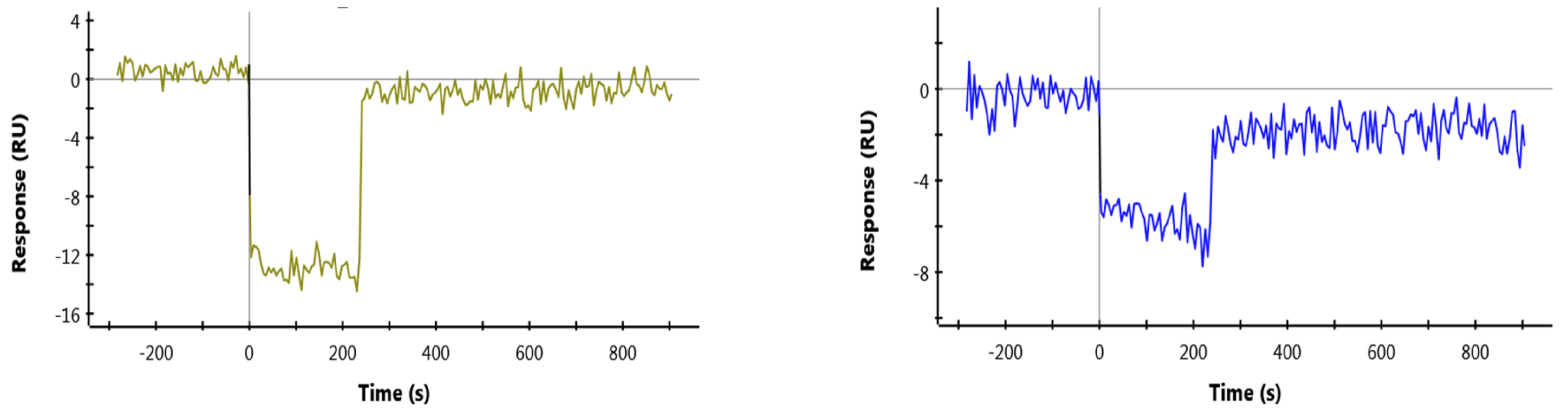
Binding of mAbs to rhFcRn and FcγRIIIa-Phe. Binding sensograms of (**a**) wild type IgG1, (**b**) ZRC3308-A7, and (**c**) ZRC3308-B10 to rhFcRn. Binding sensograms of (**d**) wild type IgG1, (**e**) ZRC3308-A7 (10 µM), and (**f**) ZRC3308-B10 (10 µM) to rhFcγRIIIa-Phe.

ZRC3308 mAbs have been designed to carry mutations in the Fc backbone to improve their FcRn binding and to reduce the binding of antibodies to rhFcγRIIIa-Phe. The ZRC3308-A7 and ZRC3308-B10 antibodies that carry the LS mutation in the Fc backbone showed ~10-fold higher affinity for binding to rhFcRn as compared to the wild type IgG1 (Figure 2a–c and Table 1). The antibodies showed no detectable binding with rhFcγRIIIa-Phe when compared to the wild type IgG1 (modified ZRC-3308 mAb with wild type Fc), indicating that ZRC3308-A7 and B10 antibodies are expected to show negligible NK-cell-mediated effector functions (Figure 2d–f and Table 1). No binding of ZRC3308-A7 and ZRC3308-B10 to the rhFcγRIIIa-Phe receptor was observed even at a very high mAb concentration of 10 µM ((Figure 2e,f). C1q binding in terms of effective concentration of mAb leading to 50% maximal binding (EC_50_) was assessed. ZRC3308-A7 (EC_50_ = 20.55 µg/mL) and ZRC3308-B10 (EC_50_ = 46.12 µg/mL) showed reduced binding when compared to wild type IgG1 (EC_50_ = 3.18 µg/mL).

**Table 1:** Binding of ZRC3308 to RBD, S trimer protein, rhFcRn, and FcγRIIIa-Phe. The kinetic rate constants of ZRC3308-A7, ZRC3308-B10, and ZRC3308 cocktail binding.

Antibody	RBD					S Trimer				
	Ka	Kd	KD	SD	%CV	Ka	Kd	KD	SD	%CV
	1/Ms	1/s	M			1/Ms	1/s	M		
ZRC3308-A7 mAb (*n* = 4)	4.57 × 10^6^	5.42 × 10^−4^	1.19 × 10^−10^	2.38 × 10^−12^	2.01	2.80 × 10^6^	4.53 × 10^−5^	1.64 × 10^−11^	2.98 × 10^−12^	18.24
ZRC3308-B10 mAb (*n* = 4)	4.05 × 10^6^	6.39 × 10^−4^	1.60 × 10^−10^	2.45 × 10^−11^	15.36	2.73 × 10^6^	3.49 × 10^−5^	1.30 × 10^−11^	2.89 × 10^−12^	22.29
ZRC3308 cocktail (1:1) (*n* = 4)	2.93 × 10^6^	5.41 × 10^−4^	1.85 × 10^−10^	2.00 × 10^−11^	10.81	1.47 × 10^6^	3.73 × 10^−5^	2.60 × 10^−11^	5.23 × 10^−12^	20.15
	**rhFcRn**					**rhFcγRIIIa-Phe**				
Wild type IgG1 (*n* = 2)	9.86 × 10^5^	1.92E × 10^−1^	1.95 × 10^−7^	2.83 × 10^−9^	1.45	3.85 × 10^4^	6.12 × 10^−2^	1.62 × 10^−6^	2.76 × 10^−7^	17.08
ZRC3308-A7 mAb (*n* = 4)	1.68 × 10^6^	2.28 × 10^−2^	1.36 × 10^−8^	6.85 × 10^−10^	5.03	NB	NB	NB	-	-
ZRC3308-B10 mAb (*n* = 4)	1.01 × 10^6^	3.42 × 10^−2^	3.40× 10^−8^	3.03 × 10^−9^	8.90	NB	NB	NB	-	-

NB: No binding.

### 3.3. Virus Neutralizing Ability of ZRC3308 mAbs

**Figure 3:**
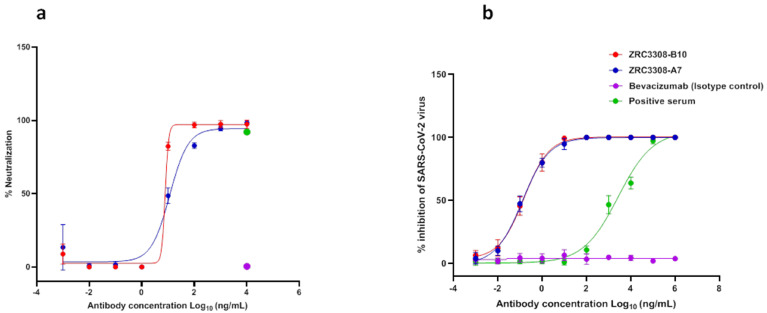
Neutralization potential of mAbs of the ZRC3308 cocktail. (**a**) The percent neutralization relative to virus control against the serial dilutions of ZRC3308-A7 and ZRC3308-B10, Bevacizumab (isotype control) and positive serum in the pseudovirus assay. (**b**) The percent inhibition of SARS-CoV-2 against serial dilutions of ZRC-3308A7, ZRC-3308B10, Bevacizumab (isotype control), and positive serum. Data are plotted as a mean ± standard deviation of four determinants.

The neutralizing activities of antibodies were confirmed by both infection of pseudotyped luciferase lentivirus in the HEK293 ACE2-expressing cells and live SARS-CoV-2 in VeroE6/Vero CCL81 cells. The mAbs of the ZRC3308 cocktail exhibited potent neutralization activity against pseudotyped luciferase lentivirus, with an IC_50_ of 12.53 and 7.78 ng/mL for ZRC3308-A7 and ZRC-3308-B10, respectively (Figure 3a). There was neutralization of pseudotyped lentivirus for the positive serum at the concentration tested, while no pseudotyped virus neutralization was observed for the isotype control. The live virus plaque reduction neutralization test (PRNT) using SARS-CoV-2 B.1 variant also showed potent neutralization activity in sub picomolar range with an IC50 of 0.1527, 0, and 0.1283 ng/mL for ZRC3308-A7 and ZRC-3308-B10, respectively. There was a dose dependent neutralization of SARS-CoV-2 with the positive serum, while no SARS-CoV-2 neutralization was obtained with the isotype control (Figure 3b). The ZRC3308 cocktail showed PRNT_50_ titers of 411781, 327205, 456261, and 456261 against B.1.1.7, B.1.351, B.1.617.2, and B.1.617.2 AY.1 variants, respectively.

### 3.4. Pharmacokinetic Study in Syrian Hamsters

Considering the tissue bioavailability and PRNT_50_ data, we performed a pharmacokinetic study with doses of 50, 5, and 1 mg/kg doses of ZRC3308 cocktail in 1: 1 ratio in Syrian hamsters for a period of 7 days. A dose-dependent increase was observed with all the doses of ZRC3308 cocktail in maximum concentration (C_max_), area under curve (AUC) _last_, and AUC _inf-obs_ of the mAb in the serum (Supplementary Table S1). The serum levels of the mAbs remained constant without much reduction for up to seven days (Supplementary Figure S1).

### 3.5. ZRC3308 mAb Prophylaxis in Syrian Hamsters and Virus Challenge

**Figure 4:**
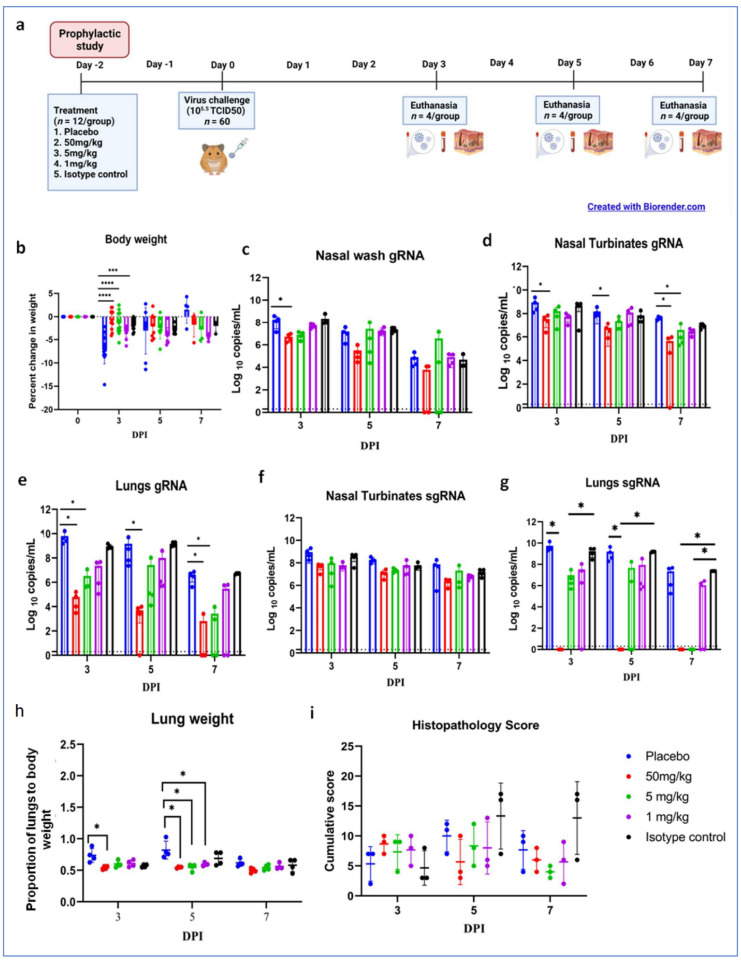
Prophylactic mAb treatment in hamsters. (**a**) Study design of the prophylactic study, (**b**) percent bodyweight change in hamsters on 3 (*n* = 12/group), 5 (*n* = 8/group), and 7 (*n* = 4/group) DPI. Viral gRNA load in (**c**) nasal wash, (**d**) nasal turbinates, and (**e**) lungs post infection. Viral sgRNA load in (**f**) nasal turbinates and (**g**) lungs post infection. (**h**) Lung weight to body weight proportion of hamsters at necropsy. (**i**) Cumulative score of lung pathological changes on 3, 5, and 7 DPI. Mean ± SD is plotted on the graph, and comparison was performed between the treated groups and the control groups. The Kruskal–Wallis test followed by Mann–Whitney test was used to assess statistical significance. An asterisk indicates a significant difference between the means, with ****, ***, and * representing *p* < 0.0001, *p* < 0.001, and *p* < 0.05, respectively, and the dotted line on the graph indicates the assay limit of detection.

For the study, hamsters (*n* = 12/group) were treated with the ZRC3308 cocktail (50, 5, and 1 mg/kg ZRC3308 cocktail) 48 h prior to the SARS-CoV-2 infection (Figure 4a). No clinical signs were observed following the mAb cocktail treatment and virus infection. Body weight was significantly reduced in the placebo group (mean ± standard deviation (SD) = −7.06 ± 3.13%) compared to 50 (mean ± SD = −0.73 ± 2.1%, *p* < 0.0001), 5 (mean ± SD = −1.44 ± 2.42%, *p* < 0.0001), and 1 mg/kg (mean ± SD = −3.2 ± 1.24%, *p* < 0.0005) treatment groups on three days post-infection (DPI) (Figure 4b). On further days, even though body weight reduction was there, it was not significant. A sustained level of mAbs in serum without much decrease was observed during the study period (Supplementary Table S2). Viral genomic RNA (gRNA) and subgenomic RNA (sgRNA) levels in the upper and lower respiratory tract showed a decreasing trend in all the mAb-treated groups until 7 DPI (Figure 4c–g). The viral load was significantly reduced in the nasal turbinates (*p* < 0.05) and lungs (*p* < 0.05) in the 50 mg/kg dose group on 3, 5, and 7 DPI compared to the placebo group. Other dose groups, i.e., 5 and 1 mg/kg groups, also showed a reduction in the viral gRNA load in lungs compared to placebo, although it was not statistically significant. Viral sgRNA could not be detected in the lungs of the 50 mg/kg dose group from 3DPI (*p* < 0.05).

**Figure 5:**
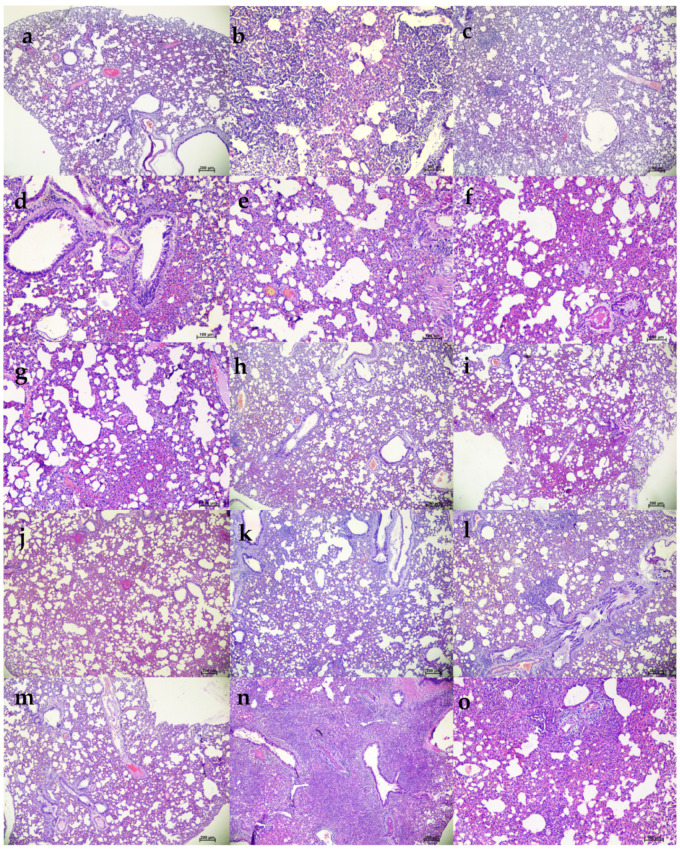
Histopathological observations in lungs of hamsters treated with ZRC3308 cocktail prophylactically. Lungs of the placebo group (**a**) on 3DPI showing congestion and focal area of consolidation, scale bar = 200 µm (**b**) on 5 DPI showing diffuse areas of consolidation, congestion, and mononuclear infiltration, scale bar = 100 µm (**c**) on 7 DPI showing congestion and consolidation, scale bar = 200 µm. Lungs of 50 mg/kg dose prophylactic group (**d**) on 3DPI showing alveolar capillary congestion, scale bar = 100 µm (**e**) on 5DPI and (**f**) 7DPI showing alveolar septal thickening and congestive changes, scale bar = 100 µm. Lungs of 5 mg/kg dose group on (**g**) 3 DPI, scale bar = 200 µm (**h**) 5 DPI and (**i**) 7 DPI showing congestive changes, scale bar = 200 µm. Lungs of 1 mg/kg dose group on (**j**) on 3DPI showing congested vessels, (**k**) on 5DPI and (**l**) 7DPI showing congestion and foci of mononuclear cell infiltration in the peri bronchial region, scale bar = 200 µm. Lungs of the isotype antibody control group on (**m**) 3 DPI showing severe congestion, scale bar = 200 µm (**n**) on 5DPI showing diffuse pneumonic changes, scale bar = 200 µm and (**o**) on 7 DPI showing alveolar septal thickening and consolidation, scale bar = 200 µm.

Grossly, mild congestive changes were observed in lungs in all the mAb-treated and control groups on 3 DPI. On the fifth DPI, congestion and hemorrhagic changes were more prominent in the 1 mg/kg dose group, isotype antibody, and placebo groups. Lungs of the 50 and 5 mg/kg groups appeared normal or with minimal gross changes on 5 and 7 DPI. The proportion of lungs’ weight to body weight in hamsters was also significantly higher in the placebo group (*p* < 0.05) on 5 DPI compared to the mAb administered group (Figure 4h). On histopathological examination, the 50 mg/kg and 5 mg/kg dose groups showed mild changes on 3, 5, and 7 DPI (mild alveolar consolidation, focal septal thickening, and inflammatory cell infiltration), whereas the 1 mg/kg group and placebo group showed moderate lesions (diffuse exudative changes in the alveolar lumen, septal thickening, and marked inflammatory cell infiltration in the interstitial space and in the peri bronchial region). The isotype antibody control showed severe pneumonic changes of diffuse involvement compared to other groups (Figure 4e and Figure 5).

### 3.6. ZRC3308 mAb Therapy in Syrian Hamsters Following the SARS-CoV-2 Challenge

**Figure 6:**
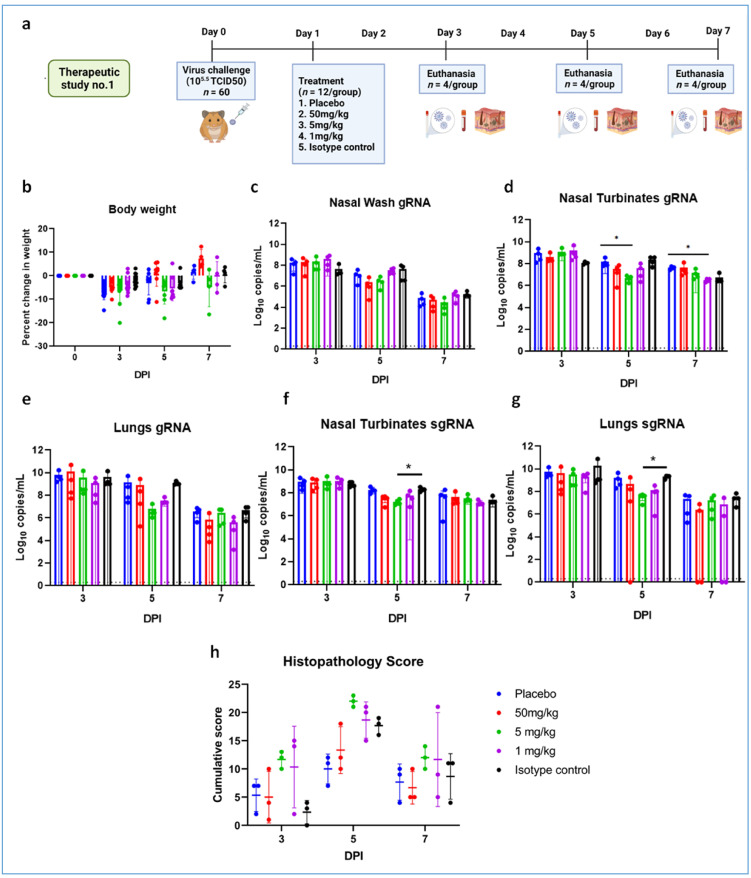
mAb therapy at 24 h post high virus dose challenge in hamsters. (**a**) Study design, (**b**) percent bodyweight gain in hamsters on 3 (*n* = 12/group), 5 (*n* = 8/group), and 7 (*n* = 4/group) DPI. Viral genomic RNA load (*n* = 4/group) in (**c**) nasal wash, (**d**) nasal turbinates, and (**e**) lungs post infection. Viral sub genomic RNA load in hamsters’ (**f**) nasal turbinates and (**g**) lungs post infection. (**h**) Cumulative score of lung pathological changes in hamsters (*n* = 3/group) post virus challenge. Mean ± SD is plotted on the graph, and a comparison was performed between the treated groups and the control groups. A Kruskal–Wallis test followed by Mann–Whitney test was used to assess statistical significance. An asterisk indicates a significant difference between the means, with * representing *p* < 0.05, respectively, and the dotted line on the graph indicates the assay limit of detection.

We studied the therapeutic potential with 10^5.5^ TCID50 (high virus dose) at 24 h (Figure 6a) and 6 h post challenge and with 10^3.5^ TCID50 (low) virus dose at 24 h after infection. In the 10^5.5^ TCID50-challenged (at 24 h) group, no significant difference could be observed with body weight loss among groups, whereas gain in body weight was observed in the 50 mg/kg group (Figure 6b). The mAb levels in the serum remained constant from Day 3 to Day 7 without much drop (Supplementary Table S2). Although gRNA and sgRNA levels in the respiratory tract showed a decreasing trend in all the groups until 7DPI, there was no statistical significance in comparison to control in the viral load (Figure 6c–g). Grossly pneumonic changes were observed in all groups with comparable lung weights to that of placebo group. On histopathological examination, moderate to severe pneumonic changes were observed in all treated and control groups (Figure 6h, Supplementary Figure S2).

**Figure 7:**
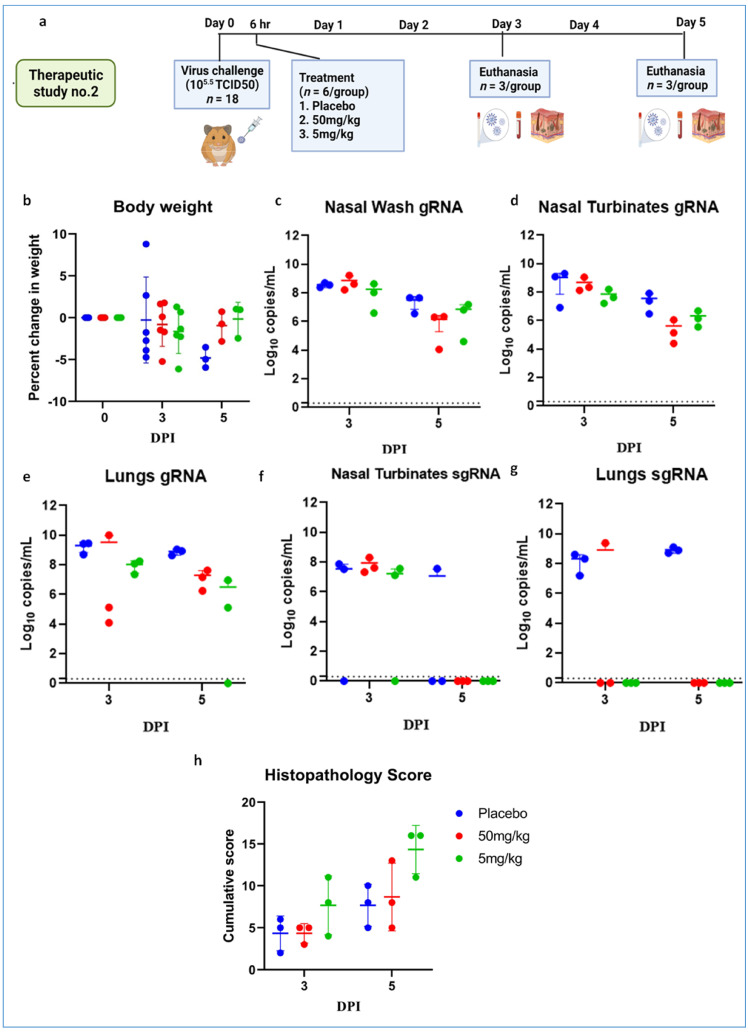
mAb therapy at 6 h following high virus dose challenge in hamsters. (**a**) Study design, (**b**) percent bodyweight gain/loss in hamsters on 3 (*n* = 6/group) and 5 (*n* = 3/group) post SARS-CoV-2 infection. Viral gRNA load (*n* = 3/group) in (**c**) nasal wash, (**d**) nasal turbinates, and (**e**) lungs in hamsters post virus challenge. Viral sgRNA load (*n* = 3/group) in (**f**) nasal turbinates and (**g**) lungs in hamsters post virus challenge. (**h**) Cumulative histopathological score of lung pathological changes in hamsters (*n* = 3/group) on 3 and 5 DPI. Mean ± SD is plotted on the graph, and a comparison was performed between the treated groups and the control groups. A Kruskal–Wallis test followed by a Mann–Whitney test was used to assess statistical significance. The dotted line on the graph indicates the assay detection limit.

Further, we performed therapeutic evaluation of the mAb cocktail (50 and 5 mg/kg) at 6 h post virus (10^5.5^ TCID50) infection to understand the ability of mAb to neutralize at a shorter interval treatment (Figure 7a). The body weight reduction was observed in both the treated groups (Figure 7b). Although the placebo group showed considerably higher body weight loss than the mAb-treated groups, it was not statistically significant. The average gRNA load in nasal wash, nasal turbinate, and lungs on 3 and 5 DPI did not show any significant difference among groups (Figure 7c–e). SgRNA levels showed reduction in comparison to the placebo group and were completely cleared from nasal turbinates and lungs by 5 DPI in all mAb-treated groups (Figure 7f,g). On histopathological examination, mild changes were seen in the 50 mg/kg dose group, whereas the hamsters of the 5 mg/kg group showed to mild to moderate changes, and the placebo group showed moderate pneumonic changes (Figure 7h, Supplementary Figure S3).

**Figure 8:**
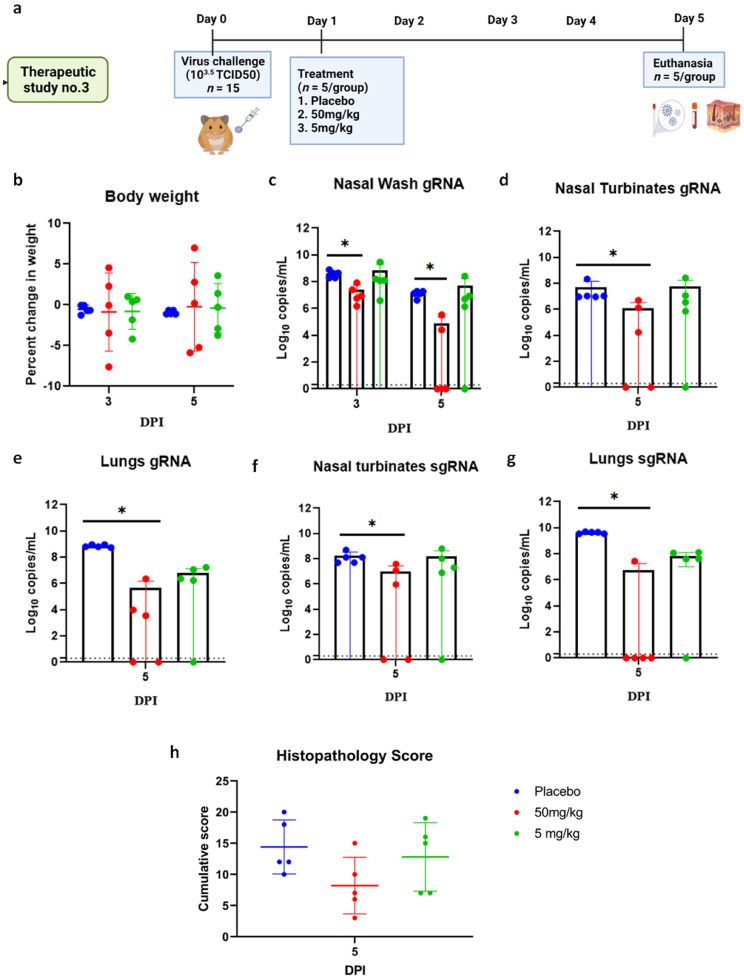
mAb therapy at 24 h post infection with 10^3.5^ TCID50 virus dose challenge in hamsters. (**a**) Therapeutic study design. (**b**) Percent bodyweight gain/loss in hamsters on 3 (*n* = 5/group) and 5 (*n* = 5/group) DPI. Viral gRNA load (*n* = 5/group) in (**c**) nasal wash, (**d**) nasal turbinates, and (**e**) lungs in hamsters post challenge. Viral sgRNA load (*n* = 5/group) in (**f**) nasal turbinates and (**g**) lungs in hamsters post infection. (**h**) Cumulative histopathological score of lung pathological changes in hamsters (*n* = 5/group) post virus challenge. Mean ± SD is plotted on the graph, and comparison was performed between the treated groups and the control groups. A Kruskal–Wallis test followed by Mann–Whitney test was used to assess statistical significance. An asterisk indicates significant difference between the means, with * representing *p* < 0.05, and the dotted line on the graph indicates the assay limit of detection.

**Figure 9:**
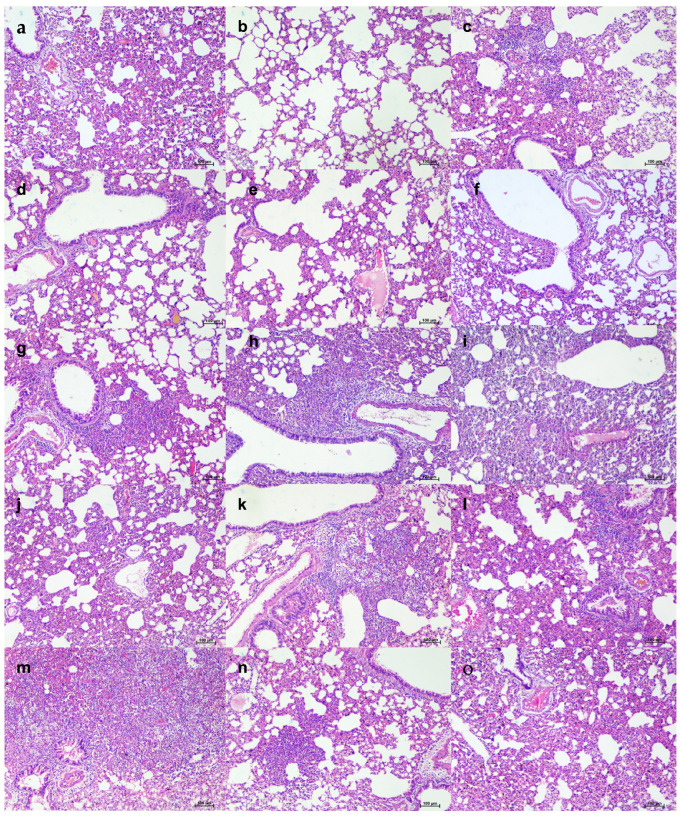
Histopathological changes in lungs of hamsters that received mAb therapy 24 h post infection with 10^3.5^ TCID50. Lungs of hamsters of 50 mg/kg dose group on 5DPI showing (**a**) engorged alveolar capillaries, (**b**) emphysematous changes, (**c**) consolidation and mononuclear cell infiltration, (**d**) foci of alveolar capillary congestion, and (**e**) alveolar septal thickening, scale bar = 100 µm. Lungs of hamsters of the 5 mg/kg dose group on 5DPI showing (**f**) focal areas of congestion and septal thickening, (**g**) peribronchial inflammatory cell infiltration and foci of consolidative changes, (**h**) diffuse inflammatory cell infiltration in the alveolar paranchyma, (**i**) diffuse alveolar changes and congestion, and (**j**) diffuse alveolar septal thickening, scale bar = 100 µm. Lungs of placebo group on 5DPI showing (**k**) diffuse inflammatory cell infiltration in the peribronchial area, (**l**) peribronchial inflammatory cell infiltration and alveolar septal thickening, (**m**) diffuse alveolar consolidation and inflammatory cell infiltration, (**n**) foci of mononuclear cell infiltration and septal thickening, and (**o**) diffuse alveolar septal thickening and congestive changes, scale bar = 100 µm.

In the 10^3.5^ TCID50 virus dose group, only minimal bodyweight reduction was seen in the 50 and 5 mg/kg mAb-treated group (Figure 8a,b). The 50 mg/kg group showed consistent viral load reduction on 3 and 5 DPI in nasal wash (*p* < 0.05), lungs (*p* < 0.05) and nasal turbinates (*p* < 0.05) (Figure 8c–e). SgRNA levels in the lungs (*p* < 0.05) and nasal turbinates (*p* < 0.05) also were significantly lower compared to the placebo group (Figure 8f,g). On histopathological examination, mild to moderate changes were seen in the 50 mg/kg dose group with a lower histopathological score, whereas the hamsters of the 5 mg/kg group and placebo group showed comparable disease severity (Figure 8h and Figure 9).

## 4. Discussion

The COVID-19 pandemic shows no signs of subsiding, as is evident from the repeated resurgence even after immunization of the majority of the population in some countries. Vaccines against SARS-CoV-2 have been rolled out as prophylactic interventions, but the treatment options are still very limited. mAbs appear to be promising candidates for SARS-CoV-2, as is evident by the EUA received by 3 mAb therapies from USFDA, which have also been granted approvals in other countries [15,16,17]. The ZRC3308 mAbs showed good binding affinity, similar to other mAb products for treatment for SARS-CoV-2 [16]. As these antibodies bind non-competitively to the RBD of the spike protein and are equipotent in terms of binding to RBD and virus neutralization potential, the 1:1 cocktail diminishes the ability of variants to escape the treatment. In vitro PRNT against variants also demonstrated the cross-protection. FcγRIIIa, a fragment crystallizable (Fc) receptor (FcR) present on NK cells, binds to IgG and mediates antibody-dependent cellular cytotoxicity (ADCC). FcγRIIIa-expressing NK cells are the key innate lymphocytes that represent the major subset in the peripheral blood (≈90%) [21,22]. The mutations designed to reduce the immune effector functions of the ZRC3308 mAbs led to a marked reduction in C1q-binding, indicating reduced complement-dependent cytotoxicity function and no detectable binding to rhFcγRIIIa-Phe in SPR, further indicating negligible NK cell-mediated ADCC. ZRC3308 mAbs are designed to have increased serum half-life, which was demonstrated in hamsters where t1/2 was found to be more than 7 days. The increased serum half-life is also reported to be associated with improved lung bioavailability [23].

We have used a Syrian hamster model to evaluate the protective efficacy of the mAb cocktail. The model has the advantages of exhibiting overt clinical signs such as body weight loss, replicating to high virus titers in the respiratory tract and severe pneumonia following SARS-CoV-2 infection, which can be used as criteria to evaluate countermeasures [24,25]. Viral load reduction is also used as a criterion to evaluate the effect of mAb in the magnitude of infection in human clinical trials [26,27]. Several research groups have evaluated mAbs for SARS-CoV-2 for their protective efficacy in the hamster model [28,29,30,31,32,33,34]. SARS-CoV-2 produces severe lung disease in the hamster model within a short incubation period of 3–5 days [24,25]. The disease severity in hamsters depends on the inoculum dose, and the pneumonic changes will set in too rapidly at high virus doses in hamsters, making it difficult to demonstrate therapeutic efficacy in the model [28,29,30]. We included two more sub studies with shorter time intervals for treatment considering the replication cycle length of SARS-CoV-2 and also with a lower virus challenge dose.

Prophylactic administration of the ZRC3308 cocktail at a high dose caused significant reduction in the lungs sgRNA and protected hamsters from developing pneumonia. In an earlier study where the REGN-COV2 prophylactic efficacy was studied in hamsters, no significant reduction could be observed in the lung viral load on seventh day post-infection, even though there was a reduction in the average viral gRNA load [28]. Although REGN-COV2 could cause prevention of body weight loss therapeutically at 50 mg/kg dose rate in hamsters, the gRNA and sgRNA loads were found to be non-significant compared to the placebo control. We also observed similar results of no reduction in gRNA load and body weight gain with our mAb cocktail following treatment at 24 h post infection with a high virus dose. Antibody-dependent virus uptake can effectively provide viral clearance but, due to its effector functions, could cause cytokine release, resulting in inflammation and tissue destruction. In the case of COVID-19, a small delay in treatment may lead to a significantly higher number of host cells becoming infected, which makes it difficult to demonstrate the benefit of mAb in severe COVID-19 cases, where inflammation and coagulopathy induce more damage than viral replication [27]. Anti-SARS-CoV-2 mAbs, which received EUA such as bamlanivimab plus etesevimab and casirivimab plus imdevimab, have also not been shown to be beneficial in hospitalized patients with severe COVID-19 and these products warn of the worst clinical outcomes in such patients [16,17]. The mAbs in an antigen-specific fashion may bind to the SARS-CoV-2-infected tissues that are expressing the viral spike protein on their cell surface. Such binding may lead to tissue damage via the mAb’s immune effector functions. The trials have also shown low clinical benefits with LY-CoV555 treatment in hospitalized COVID-19 patients [27]. We also observed severe pneumonic changes in the group infected with a high inoculum dose treated with mAb 24 h post-infection. However, the similar changes were not observed in the prophylactic and therapeutic groups exposed with a lower virus dose. Rapid induction of pneumonic changes in the hamster model is a limitation of the therapeutic evaluation of this model. Studies that have successfully used this model for therapeutic evaluation have used a smaller time interval for treatment post infection or with a lower virus dose [31,35]. We observed reduction in viral load following mAb treatment in our lower virus dose group and with a shorter interval treatment group. However, the 6 h therapeutic group had the limitation of a small sample size. The severity of pneumonia observed in the placebo group of the 6 h therapeutic group was higher, which could be due to the more aged animals used in this substudy. Increased weight loss and severe histopathology have been reported in the aged hamsters following SARS-CoV-2 infection [36]. In another preclinical study of a human monoclonal antibody AvGn-B in Syrian hamsters, lung disease severity was reduced following the intraperitoneal antibody treatment post-infection. In this study, the virus dose used to infect Syrian hamsters was similar to the lower virus dose used in our present study [31]. The reduction in the viral sgRNA in the nasal wash and nasal turbinates was also observed here in the mAb-treated animals, which is important as the upper respiratory tract viral load is a key determinant of transmission. The interim results of the Phase 1–3 clinical trials (ClinicalTrials.gov number: NCT04425629) of the mAb REGN-COV2 in patients with early infection showed a reduced SARS-CoV-2 RNA level in nasopharyngeal swab samples following mAb administration [37]. Similar results were reported in the case of bamlanivimab (LY-CoV555) treatment in patients with a median of 4 days after symptom onset [38].

The route of administration is also an important factor affecting the lung bioavailability of mAb. Intravenous administration of the mAbs can result in suboptimal lung availability [32,33]. The mAb STI-2020, when studied in mice for the biodistribution by intranasal and intravenous routes, showed lower lung availability via the intravenous route within 24 h post administration [33]. The dose-sparing effects of the local administration of mAbs for SARS-CoV-2 treatment have been demonstrated in Syrian hamsters [32,34]. The 1212C2 mAb delivery via the inhalation route showed superior therapeutic efficacy in hamsters when compared with parenteral administration, where the percentage of delivered mAb dose detected in the bronchioalveolar lavage after intraperitoneal administration was very low [34]. However, we have not assessed the lung availability of the mAb cocktail following the treatment. The similar serum antibody concentration in the blood in the therapeutic group to that of prophylactic group at 3DPI could not prevent the disease progression in the former. Monoclonal antibodies are commonly associated with self-immunogenicity, which may lead to tissue damage and other treatment-related toxicity. A four-week repeat dose toxicology study was carried out in rhesus monkeys and wistar rats (up to 220 mg/kg), and no treatment-related adverse reactions or concerns were observed upon gross and histopathological evaluation, indicating minimal or no tissue cross reactivity of the ZRC-3308 cocktail (data not shown).

In the present study, we have characterized the ZRC 3308 mAb cocktail for COVID-19 treatment, which was found to be cross-neutralizing and a promising candidate for the prophylactic use and for therapy in early cases that have not progressed to severe disease. The product appears to be a promising candidate to be taken up to the clinical trials.

## Data Availability

The data presented in this study are available in the article and Supplementary Materials.

## Supplementary Materials

The following are available online at https://www.mdpi.com/article/10.3390/v13122424/s1, Figure S1: Pharmacokinetic study of ZRC3308 cocktail in Syrian hamsters, Figure S2: Histopathological changes in lungs of hamsters which received mAb therapy 24 h post infection, Figure S3: Histopathological changes in lungs of hamsters which received mAb therapy 6 h post infection, Table S1: Pharmacokinetic parameters of ZRC3308 cocktail, Table S2: Serum concentration of monoclonal antibody post virus infection at 3, 5 and 7 days.
