# Supplementary material for: ZRC3308 Monoclonal Antibody Cocktail Shows Protective Efficacy in Syrian Hamsters against SARS-CoV-2 Infection
Source: Viruses. 2021 Dec 3;13(12):2424. doi: 10.3390/v13122424 (PMC8706527; doi:10.3390/v13122424)
Supplement: Supplementary file 1 [file viruses-13-02424-s001.zip › viruses-1421467-supplementary.pdf]

Supplementary data

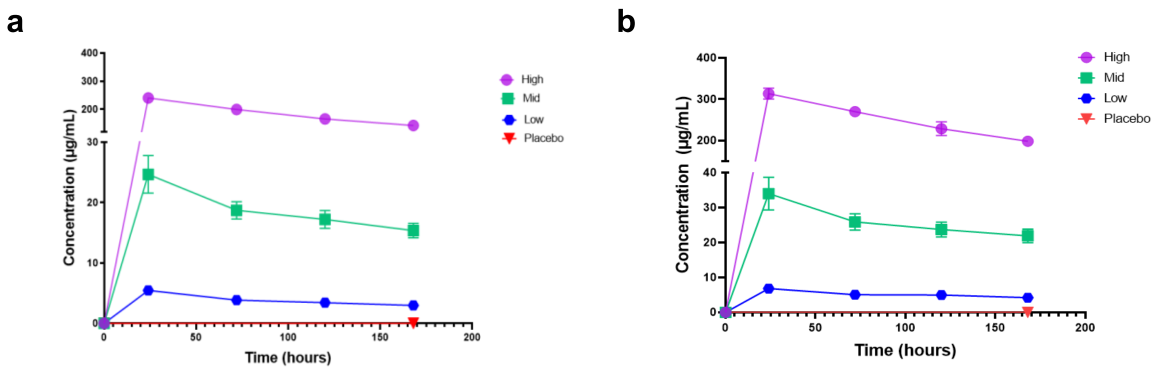

Supplementary Figure S1: Pharmacokinetic study of ZRC3308 cocktail in Syrian hamsters. a. Pharmacokinetic profile of ZRC3308-A7 in hamster serum for the 50 mg/kg, 5 mg/kg, 1 mg/kg and placebo. **b.** Pharmacokinetic profile of ZRC3308-B10 in hamster serum for the 50 mg/kg, 5 mg/kg, 1 mg/kg and placebo.

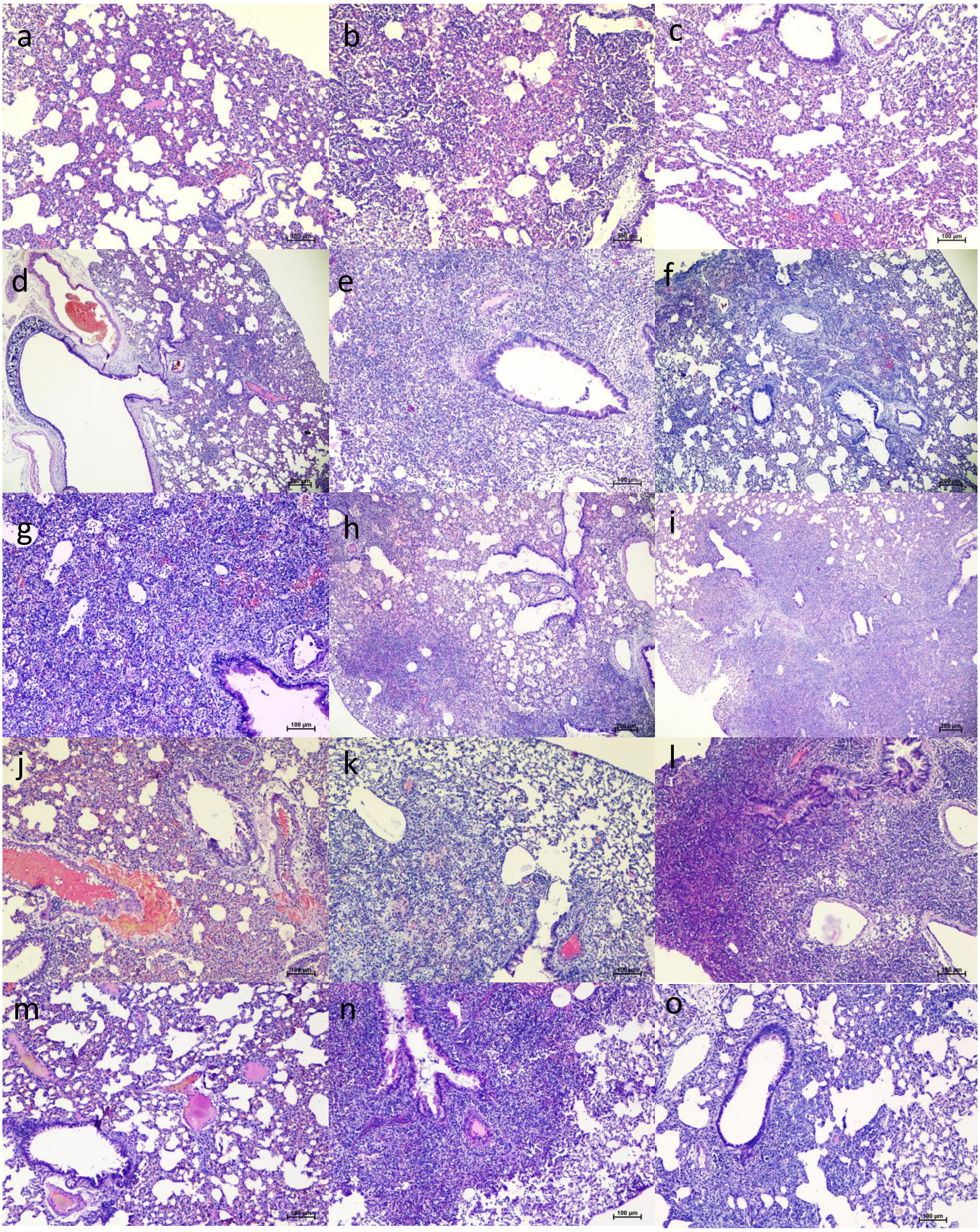

Supplementary Figure S2: Histopathological changes in lungs of hamsters which received mAb therapy 24 hours post infection. Lungs of placebo group **(a)** on 3DPI showing congestion and focal area of consolidation, **(b)** on 5 DPI showing extensive areas of congestion septal thickening and **(c)** on 7 DPI showing consolidation and congestion. Lungs of 50 mg/kg dose prophylactic group **(d)** on 3DPI showing congestion and focal area of infiltration **(e)** on 5DPI showing diffuse mononuclear infiltration, pneumocyte hyperplasia and exudative changes and **(f)** on 7DPI showing peri bronchial mononuclear infiltration and haemorrhages. Lungs of 5mg/kg dose group on **(g)** 3 DPI showing haemorrhages, **(h)** 5 DPI showing alveolar exudative changes, multifocal areas of mononuclear

infiltration and **(i)** on 7 DPI showing diffuse mononuclear infiltration. Lungs of 1 mg/kg dose group on **(j)** on 3DPI showing severely congested vessels, **(k)** on 5DPI showing congestion and foci alveolar septal thickening with pneumocyte hyperplasia. and **(l)** 7DPI showing diffuse alveolar damage. Lungs of the isotype antibody control group on **(m)** 3 DPI showing severe congestion **(n)** on 5DPI showing peri bronchial mononuclear infiltration and on **(o)** 7 DPI showing infiltration in the peri bronchial area and collapse of surrounding alveoli.

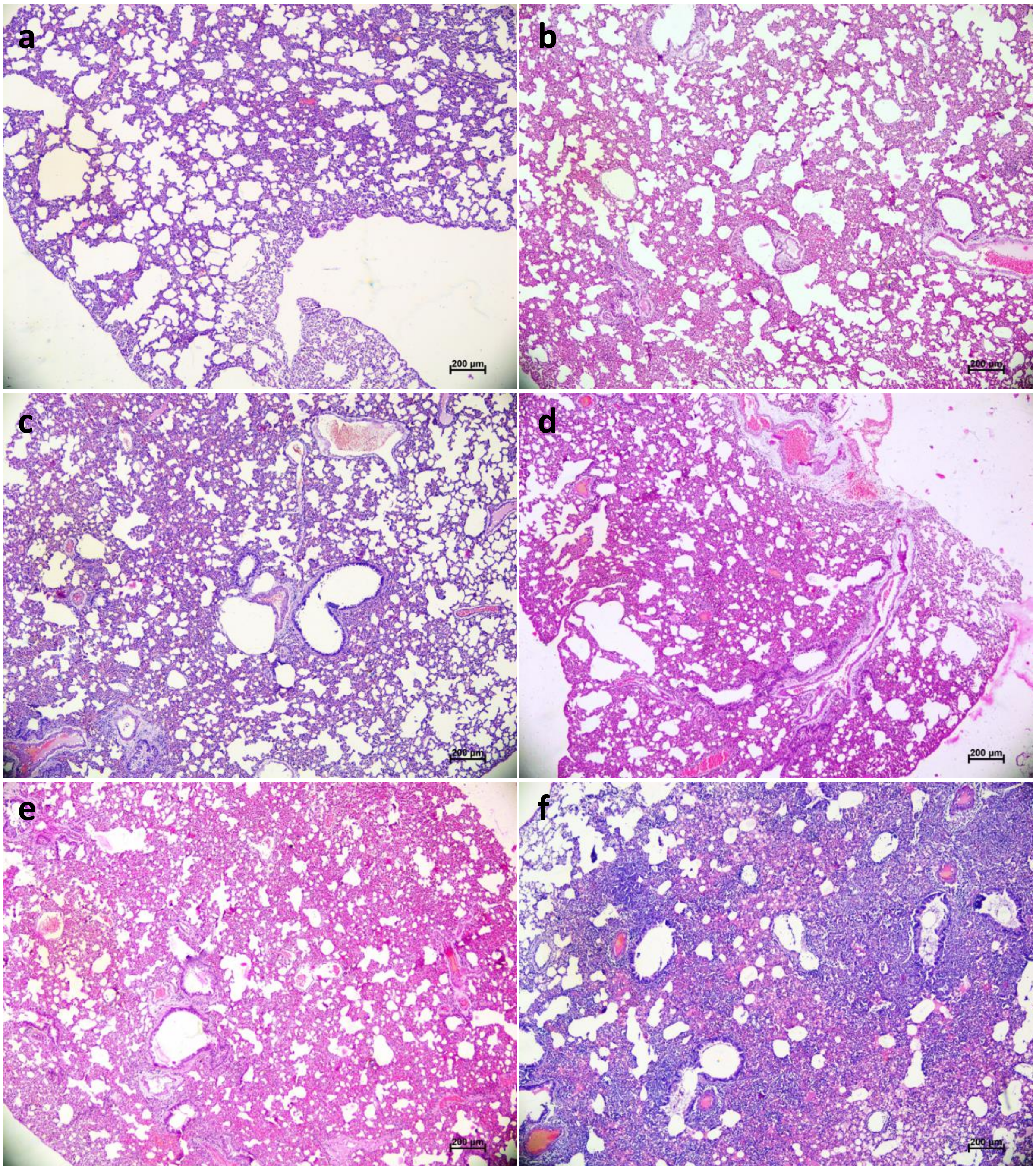

Supplementary Figure S3: Histopathological changes in lungs of hamsters which received mAb therapy 6 hours post infection. Lungs of 50mg/kg dose group **(a)** on 3DPI showing mild congestion **(b)** on 5 DPI showing congestion and atelectasis. Lungs of 5 mg/kg dose group **(c)** on 3DPI showing congestion and focal area of infiltration and **(d)** on 5DPI showing severe congestion, consolidative changes and focal mononuclear infiltration. Lungs of placebo group showing **(e)** haemorrhage,

congestion and exudative changes on 3DPI and **(f)** on 5 DPI showing diffuse mononuclear infiltration, congestion, pneumocyte hyperplasia and consolidation.

Supplementary Table S1. Pharmacokinetic parameters of ZRC3308 cocktail.
Thepharmacokinetic profile of ZRC-3308A7 and ZRC-3308B10 mAbs in the hamster serum at T_{max} of 24 hours.

**Dose (mg/kg)**		**ZRC3308-A7**			**ZRC3308-B10**		
		**C_{max} (µg/mL)**	**AUC_{last} (h*µ/mL)**	**AUC_{ifn_obs} (h*µg/mL)**	**C_{max} (µg/mL)**	**AUC_{last} (h*µ/mL)**	**AUC_{ifn_obs} (h*µg/mL)**
**0.5**	N	4	4	4	4	4	4
	Mean	5.47	619.29	1470.99	6.82	828.74	2226.37
	SD	0.54	29.84	187.91	0.76	42.72	286.69
**2.5**	N	5	5	5	5	5	5
	Mean	24.67	2983.02	8521.71	33.99	4130.48	14129.56
	SD	3.11	234.55	1714.57	4.67	360.43	3277.83
**25**	N	5	5	5	5	5	5
	Mean	240.4	29624.06	72933.74	313.48	39974.5	103423.03
	SD	7.19	1258.91	13487.96	13.34	1792.82	13754.51

*T_{max} reported as median

One animal in 0.5 mg/kg has been excluded from analysis as no drug could be detected which may be due to dosing error

Supplementary Table S2: Serum concentration of monoclonal antibody post virus infection at 3, 5 and 7 days

Group	Days post infection	50mg/kg dose		5 mg/kg dose		1 mg/kg dose	
		ZRC-3308- A7 Concentration (mg/ml)	ZRC-3308-B10 Concentration (mg/ml)	ZRC-3308- A7 Concentration (mg/ml)	ZRC-3308-B10 Concentration (mg/ml)	ZRC-3308- A7 Concentration (mg/ml)	ZRC-3308-B10 Concentration (mg/ml)
Prophylactic	3	0.461	0.616	0.133	0.117	0.005	0.002
		0.368	0.422	0.139	0.127	0.006	0.002
		0.372	0.435	0.14	0.129	0.006	0.002
		0.414	0.515	0.136	0.123	0.005	0.002
	5	0.344	0.386	0.124	0.101	0.004	0.002
		0.343	0.386	0.122	0.098	0.004	0.002
		0.405	0.507	0.111	0.076	0.004	0.002
		0.369	0.443	0.122	0.1	0.004	0.002
	7	0.352	0.424	0.122	0.104	0.004	0.002
		0.369	0.448	0.12	0.108	0.004	0.002
		0.351	0.415	0.117	0.087	0.004	0.002
		0.397	0.512	0.119	0.101	0.003	0.002
Therapeutic (24 hours)	3	0.538	0.81	0.182	0.206	0.009	0.015
		0.366	0.418	0.146	0.139	0.008	0.012
		0.469	0.674	0.151	0.149	0.003	0.002
		0.494	0.669	0.141	0.132	0.008	0.011
	5	0.472	0.625	0.141	0.132	0.006	0.008
		0.512	0.722	0.13	0.11	0.006	0.009
		0.507	0.718	0.137	0.126	0.005	0.008
		0.223	0.155	0.141	0.134	0.006	0.009
	7	0.391	0.476	0.114	0.092	0.004	0.003
		0.223	0.161	0.119	0.1	0.004	0.003
		0.456	0.603	0.128	0.123	0.004	0.003
		0.431	0.614	0.118	0.098	0.004	0.004
Therapeutic (6 hours)	3	0.461	0.616	0.133	0.117		
		0.368	0.422	0.139	0.127		
		0.372	0.435	0.14	0.129		
	5	0.344	0.386	0.124	0.101		
		0.343	0.386	0.122	0.098		
		0.405	0.507	0.111	0.076		
